# Glycerine

**Published:** 1894-08

**Authors:** 


Glycerine, four parts, to bismuth, one part, is the best way to keep the latter in solution.

